# Supplementary material for: Small Cell Carcinoma of the Ovary, Hypercalcemic Type (SCCOHT) beyond SMARCA4 Mutations: A Comprehensive Genomic Analysis
Source: Cells. 2020 Jun 19;9(6):1496. doi: 10.3390/cells9061496 (PMC7349095; doi:10.3390/cells9061496)
Supplement: Supplementary file 1 [file cells-09-01496-s001.zip › cells-772761-supp-final-3/cells-772761-Suppl-final 2.pdf]

# Supplementary Material and Methods

### Patients and samples

Frozen tumors from 8 patients with SCCOHT were identified from the tumor banks of Gustave Roussy, Cochin Hospital, University Hospital Grenoble, Longjumeau University Hospital and Hopital de la Croix Rousse. Central review for histological diagnosis and assessment of tumor cellularity was conducted by an expert pathologist. Matched blood was available for 6 patients. All patients provided written informed consent allowing use of their tumor and healthy tissues for research. Approval from the hospital's institutional review board was obtained for the study and funding was obtained via an educational grant awarded by the Foundation Gustave Roussy.

**DNA extraction:** DNA was extracted from frozen tumors and matched blood using the AllPrep DNA Mini Kit (Qiagen, Valencia, California, USA) according to the manufacturer's instructions. Quantity was assessed by Qubit (Life Technologies, Saint-Aubin, France). The quantity of the extracted DNA was evaluated using a Qubit spectrophotometer from Invitrogen. DNA integrity was measured using an Agilent BioAnalyzer.

### Exome sequencing

One microgram (µg) of genomic DNA was sheared with the Covaris S2 system (LGC Genomics / Kbioscience). DNA fragments were end-repaired, extended with an 'A' base on the 3′ end, ligated with paired-end adaptors and amplified (six cycles). Exome-containing adaptor-ligated libraries were hybridized for 24 h with biotinylated oligo RNA baits, and enriched with streptavidin-conjugated magnetic beads using SureSelect V5 no UTR (Agilent). The final libraries were indexed, pooled and sequenced on Illumina HiSeq-2000 sequencer at IGR.

Sequencing was performed using Illumina HiSeq 2000 in paired-end mode with a mean depth of 100X producing 100bp reads. Reads were then mapped using BWA (V0.7.5a-r405) (PMID: 20080505) with MEM algorithm against reference genome hg19. Then, analysis of coverage was done using GATK (2.7.4-g6f46d11) (PMID: 20644199). DepthOfCoverage. Local realignment was performed around indels using GATK RealignerTargetCreator and GATK IndelRealigner.

Variants were called using Varscan2 (PMID: 22300766) using hg19 as the reference genome requiring a minimum tumor read depth of 6, a minimum somatic read depth of 8 and a minimum allelic frequency of 0.10. Results were then annotated using SnpEff (4.3t) (PMID: 2272867) and SnpSift (4.3t) (PMID: 22435069) with dbSNP (v150_hg19) (http://www.ncbi.nlm.nih.gov/SNP/) and dbNSFP (v2.9) (PMID: 21520341). The following further filters were applied: somatic or LOH variants; mutated allele frequency higher in tumor than normal tissue; a p-value by Fishers exact test<0.001 and variant in coding region.

A recurrence analysis was performed in order to detect which genes were mutated in at least 50% of the cohort.

Prevalence of somatic mutations (including LOH mutated) in exome was calculated based on the identified mutations in protein coding genes and divided by the size of the region processed by the variant caller (with sufficient depth of coverage) and expressed as number of mutations/megabase of DNA (mut/Mb). A Polyphen score were attributed to each mutation according to Adzhubei IA et al., 2010 (PMID: 20354512).

Mutations were validated on genomic DNA with Sanger method (GATC-biotech, FRANCE) after being amplified using Maxima SYBR/R qPCR KIT (Fermentas-Thermofisher, Courtaboeuf, FRANCE) on a ViiA 7 (Applied Biosystem / Life Technologies, Saint Aubin, France). Primers are listed in **Supplementary table S11**.

### Oligonucleotide CGH microarrays

*DNA Labeling and Hybridization*: DNA extracted from frozen samples was used to perform the CGH arrays. CGH arrays were done following standard operating procedures from Agilent Technologies (Agilent Oligonucleotide Array-Based CGH for Genomic DNA Analysis G4410-90010).

Agilent SureTag DNA Labeling Kit (Agilent, 5190-3400) was used to label Tumor DNA and control DNA (Human Genomic DNA Female, G1521, Promega) with either cyanine 3 or cyanine 5. 0.5 µg of genomic DNA was used as for the labeling reaction. DNA were fragmented by a double enzymatic digestion (Alu I+ Rsa I) and checked with Bioanalyzer and DNA 7500 Kit (Agilent Technologies). Tumor DNA and control DNA were labelled by random priming with Cy5-dUTP and Cy3-dUTP, respectively. Cyanine 3 and cyanine 5 labeled samples were purified, mixed and hybridized to Agilent microarrays from the SurePrint G3 Human CGH Microarray Kit, 8x60K (Agilent, G4450A, Design ID 021924) at 65°C for 24h at 20 rpm.

*Microarray scanning, extraction and analysis*: Microarrays were scanned using an Agilent DNA Microarray Scanner (Agilent, G2505C). Agilent's Feature Extraction software (v10.7.3.1) was used to extract data from raw microarray image files in preparation for analysis. Oligonucleotide aCGH processing was performed as detailed in the manufacturer's protocol (version 4.0; http://www.agilent.com). Data were extracted from scanned images using the Feature Extraction software (v10.7.3, Agilent), along with protocol CGH_107_Sep09. Acquired signals were normalized according to their dye and local GC% content using in-house scripts under the R statistical environment (http://cran.r-project.org). Resulting log2(ratio) were segmented using the CBS (PMID: 15475419) algorithm implementation from the DNAcopy package for R. Aberration status calling was automatically performed for each profile according to its internal noise (absolute variation of log2(ratio) values across consecutive probes on the genome). All genomic coordinates were established on the UCSC *homo sapiens* genome build hg19 (PMID: 12519945).

*Hierarchical clustering* was performed under R on the segmented data, using Euclidean distances and Ward's construction method. Quantification of the differences across pairs of samples was measured as the summed amount in size of genome involved in gains and/or in losses for each comparison, and expressed as a percentage relative to the size of the whole human genome. Comparisons of subpopulations of CGH profiles were performed under R, using a Wilcoxon test applied on each genomic region defined by the segmentation. Resulting p-values were adjusted for their false-discovery rate using Benjamini-Hochberg method (PMID: 2218183). However, as there is currently no relevant method for the FDR-adjustment of p-values for aCGH data, filtering of differential regions was performed on raw p-values, and the adjusted values are given as an indication.

**RNA Sequencing**

RNA-Seq variant discovery workflow was conducted according to Broad Institute best practices. RNA-Seq raw data (between 100 and 130 millions reads paired-end 2 x 76bp per sample) were mapped against human genome (hg19) with STAR (v2.3.0) 2-pass method described by Engström et al. (PMID: 24185836) and potential duplicates were marked using Picard tools (http://picard.sourceforge.net/). Remaining reads were split into exon segments and STAR mapping qualities were reassigned in order to fit GATK (v3.2-2) IndelRealignment requirements (PMID: 20644199 and PMID: 21478889). After local realignment around indels, a base quality score recalibration (BQSR) process was applied and the variant calling step was done with HaplotypeCaller in RNA-Seq mode. Finally, the list of raw variants obtained above was filtered on Phred-scaled p-value using Fisher's exact test to detect strand bias (FS > 30.0) and Variant Confidence/Quality by Depth (QD < 2.0) values.

The workflow for RNA-Seq chimeras (fusion transcripts) discovery is composed of RNA-Seq rawdata mapping against human genome (hg19) and transcriptome with Tophat2 (v2.0.10) described by Kim et al. (PMID: 23618408) and using "--fusion-search --library-type=fr-firststrand --mate-inner-dist 175 --mate-std-dev 75" parameters. Tophat-fusion-post (PMID: 21247443) was used to filter "fusions.out" output file with the following criteria "--num-fusion-reads 2 --num-fusion-pairs 2".

RNA expression levels were obtained by mapping RNA-Seq raw data (between 100 and 130 millions reads stranded paired-end 2 x 76bp per sample) against human genome (hg19) with Tophat (v2.0.10). The transcripts assembly and expression level quantification was done with Cufflinks (v2.2.1).

**Differential expression analysis of RNA sequencing data**

Cufflinks (2.0.2) (PMID: 22383036) was used to estimate the expression values (FPKMS), and GENCODE v19 (PMID: 22955988) GTF file for annotation. Data for benign ovarian tissue were downloaded from the GTEx portal; only GTEx samples from patients <42 years old (n=5) were selected. All reads were independently aligned with STAR_2.4.0f1 (PMID: 26334920) for sequence alignment against the human genome sequence build hg19, downloaded via the UCSC genome browser [http://hgdownload.soe.ucsc.edu/goldenPath/hg19/bigZips/] and SAMTOOLS v0.1.19 (PMID: 19505943) for sorting and indexing reads. Cufflinks (2.0.2) was used to estimate the expression values (FPKMS), and GENCODE v19 GTF file for annotation. Since benign ovary tissue samples from GTEx were processed using different library prep methods, and in order to minimalize batch effect, we performed rank normalization of gene expression data (replacing each observation by its fractional rank, i.e. the rank normalized by the total number of genes). For differential expression analysis, we calculated log2foldchange from rank normalized expression values. For statistical significance, we performed Wilcox test with Benjamini and Hochberg adjustment to p-values. R (v3.3.2) and ggplot2 (2.2.1) were used for the statistical analysis.

**Immunohistochemistry**

To establish the profile of the SMARCA4 and SMARCA2 protein expression in SCCOHT (N=40) tumors of our cohort, an immunohistochemistry assay was performed SMARCA4 protein expression was also assessed in a non-SCCOHT control panel (N=20). Assays were performed using rabbit polyclonal antibody anti-BRG-1 (Santa Cruz, sc-10768) and anti-BRM (Abcam, ab15597) respectively at a dilution of 1/200 and 1/50. After paraffin removal and hydration, the slides were immersed in 10mM citrate buffer pH 6, 30 min or antigen retrieval. The antibody was incubated one hour at room temperature, and the second antibody was incubated for 30 minutes at room temperature. The streptavidin labelled streptavidin-biotin amplification method (VECTASTAIN Elite ABC Kit) was carried out for 30 minutes followed by peroxidase/diaminobenzidine substrate/chromagen. Image acquisition was performed with a Virtual Slides microscope VS120-SL (Olympus, Tokyo, Japan), 20X air objective (0.75 NA) and analyzed with OlyVia software (Olympus, Tokyo, Japan).

**Quantitative real-time polymerase chain reaction (RT-PCR) analyses**

Total RNA was isolated from 8 SCCOHT tumors cryo-conserved and Bin-67 cells line using Trizol (Invitrogen, Carlsbad, CA, USA). Random-primed cDNA was performed using (KIT). Target cDNA sequences were quantified by real-time PCR using Maxima SYBR/R qPCR KIT (Fermentas-Thermofisher, Courtaboeuf, FRANCE). Reactions were done in triplicate on a ViiA 7 (Applied Biosystem / Life Technologies, Saint Aubin, France). *SMARCA2* expression was normalized using three housekeeping genes: *YWHAZ*, *HPRT1* and *GUSB*. Primer sequences:

*SMARCA2* fwd: CGGACGATGAGACTCTGAACCAAA, rev: CGGTCCATGTCCATCCGCATAAAA)

*YWHAZ* (fwd: GAGACGGAGCTAAGAGATATCTGC, rev: TCTCTGCTTGTGAAGCATTGGG),

*HPRT1* (fwd: TGCTTTCCTTGGTCAGGCAGTA, rev: TGGGGTCCTTTTCACCAGCAA)

*GUSB* (fwd: GCAGAAACGATTGCAGGGTTTCAC, rev: ACTGCTCTAGCAGACTTTTCTGGT)

**Cell culture and cell viability assay**

BIN-67 cell line (SCCOHT cell line) was kindly gift from Pf Vanderhyden (Ottawa Hospital Research Institute, Ottawa, CANADA). Others cell lines were obtained from American Type Culture Collection (ATCC) : NCI:H1299 (ATCC® CRL5803™) (human non small lung cancer cells), NIH:OVCAR3 (ATCC® HTB161™), SKOV3 (ATCC® HTB77™) (human ovarian adenocarcinoma) and G401 (ATCC® CRL1441™) (malignant rhabdoid tumor ). Cells lines were tested and certified as mycoplasma-free. Cells were maintained in Dulbecco's modified Eagle/F12- Dulbecco's

modified Eagle's medium for BIN-67 and RPMI-1640 for others (GIBCO/ Life Technologies, Saint Aubin, France). Mediums were supplemented with 10% fetal calf serum (PAN Biotech, Aidenbach, Germany) and 1% penicillin/streptomycin (GIBCO/ Life Technologies, Saint Aubin, France; 100µg/mL) in a 37°C incubator with 5% $CO_2$ saturation.

Cell viability was determined using MTT assay. In brief, cells were seeded into 96-well plates ($1.2\times10^4$ cells/well, 100 µL working volume per well), treated with drugs at increasing concentrations, incubated for 72 hours at 37°C and collected at 0h, 24h and 72h timepoints. MTT was added to the wells and incubated for 3 hours. Cell media was replaced by DMSO and pipetted to dissolve the formed formazan crystals. Then, optical density of the colored solution was quantified at 560 nm wavelength using a microplate reader (Victor X4 Multilabel Reader: Perkin Elmer, Courtaboeuf, France). Following compounds were tested at different concentrations (1-10-100nM): Trichostatin-A (TSA) (T8552, Sigma); 5'-Azacytidine (5'dAZAC) (Sigma, A2385). Negative control for all the assays was represented by the untreated medium containing vehicle DMSO (0.1%). Viability ratios (using the 0h timepoint as reference) between treated and untreated cells at 72h were compared using a two-way ANOVA test.

**Immunoblotting**

Cells were lysed in RIPA buffer with protease and phosphatase inhibitors (Thermo Fisher Scientific) and total protein concentration was measured using the DC Protein Assay (Bio-Rad). Protein samples were resolved in SDS-PAGE, transferred onto a nitrocellulose membrane using the iBlot 2 dry blotting system (Thermo Fisher Scientific) and incubated overnight at 4°C with primary antibodies dissolved in 5% Blotting-Grade Blocker (Bio-Rad). The following primary antibodies (provider, clone, dilution) were used: BRG1 (Abcam, EPR3912, 1/1000), BRM (Cell Signaling Technoogy, D9E8B, 1/1000), BAF47 (Abcam, EPR12014, 1/5000), BAF155 (Abcam, EPR12395, 1/5000), BAF170 (Cell Signaling Technology, D8O9V, 1/10000), BAF45B (Atlas Antibodies, polyclonal, HPA049148, 1/1000), BAF53A (Abcam, EPR7443, 1/2000), BAF250A (Abcam, EPR13501, 1/1000. After 3 washes, the membrane was incubated with secondary antibody conjugated to horseradish peroxidase for 1h at room temperature. After 3 washes, signal was visualized by chemiluminescence using the Luminata Forte substrate (Thermo Fisher Scientific) and images were acquired with the ChemiDoc™ Touch Imaging System (Bio-Rad, Hercules, CA).
