# Supplementary figures and images for: Small Cell Carcinoma of the Ovary, Hypercalcemic Type (SCCOHT) beyond SMARCA4 Mutations: A Comprehensive Genomic Analysis
Source: Cells. 2020 Jun 19;9(6):1496. doi: 10.3390/cells9061496 (PMC7349095; doi:10.3390/cells9061496)

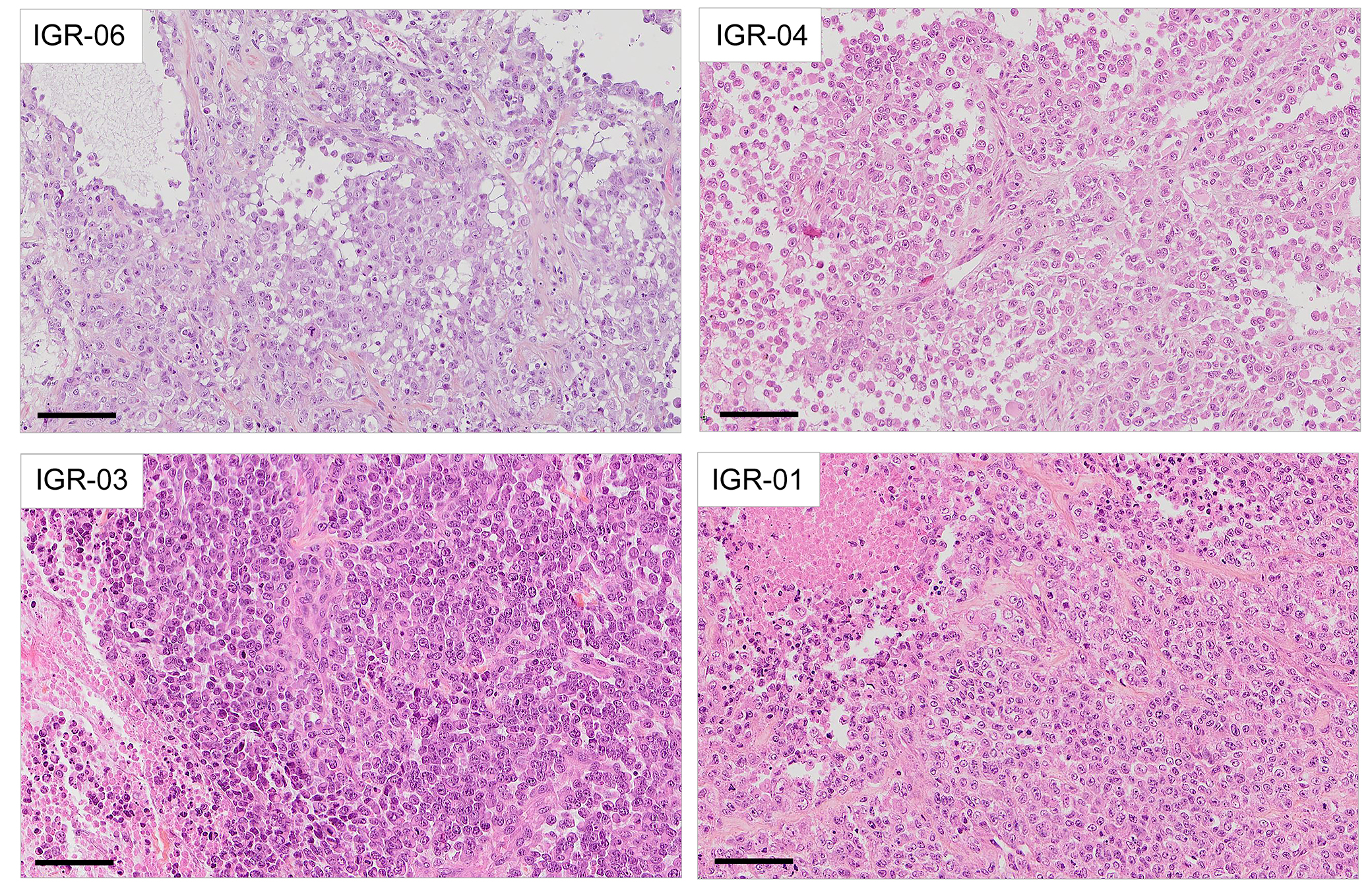

Supplement: Supplementary file 1 [file cells-09-01496-s001.zip › cells-772761-supp-final-3/Supplementary figure 1 SCCOHT.jpg]
